# Qualitative Analysis of Remineralization Capabilities of Bioactive Glass (NovaMin) and Fluoride on Hydroxyapatite (HA) Discs: An In Vitro Study

**DOI:** 10.3390/ma14143813

**Published:** 2021-07-08

**Authors:** Shu-Min Hsu, Muhammad Alsafadi, Christina Vasconez, Chaker Fares, Valentin Craciun, Edgar O’Neill, Fan Ren, Arthur Clark, Josephine Esquivel-Upshaw

**Affiliations:** 1 Department of Restorative Dental Sciences, Division of Prosthodontics, College of Dentistry, University of Florida, Gainesville, FL 32610, USA; shuminhsu@ufl.edu (S.-M.H.); MAlsafadi@dental.ufl.edu (M.A.); cvasconez@dental.ufl.edu (C.V.); EONEILL@dental.ufl.edu (E.O.); BCLARK@dental.ufl.edu (A.C.); 2 Department of Chemical Engineering, University of Florida, Gainesville, FL 32610, USA; c.fares@ufl.edu (C.F.); fren@che.ufl.edu (F.R.); 3 National Institute for Lasers, Plasma and Radiation Physics, 077125 Magurele, Romania; valentin.craciun@inflpr.ro; 4 Extreme Light Infrastructure for Nuclear Physics, IFIN-HH, 077125 Magurele, Romania

**Keywords:** hydroxyapatite, dental decay, demineralization, remineralization, bioactive glass

## Abstract

Tooth decay is a prevalent disease that initiates when the oral pH becomes acidic. Fluoride and/or bioactive glass (NovaMin) were used to regenerate/repair teeth that had been decalcified. In this present study, we investigated the effect of fluoride and/or bioactive glass (NovaMin) on remineralization of hydroxyapatite (HA) discs, which mimic the enamel surface of natural teeth. HA discs were etched with phosphoric acid and treated with one of the following toothpastes: (1) Sensodyne toothpaste with fluoride; (2) Sensodyne toothpaste with fluoride and bioactive glass (NovaMin); (3) Tom’s toothpaste without fluoride or bioactive glass (NovaMin); and (4) Tom’s toothpaste with bioactive glass (NovaMin). The toothpastes were applied on the etched discs for two minutes, once a day for 15 days. Scanning electron microscopy (SEM) was used to analyze surface morphologies and X-ray photoelectron spectroscopy (XPS) was used to analyze surface compositions. Tom’s toothpaste with only NovaMin demonstrated the most remineralization potential compared with the other groups. In conclusion, incorporating bioactive glass (NovaMin) into toothpastes could benefit the repair and remineralization of teeth.

## 1. Introduction

Dental decay is an irreversible progressive bacteriological destruction of the calcified tooth structure, and is considered a complex multifactorial process involving the interaction of four factors: host, time, diet, and microorganisms [1]. Dental caries is one of the most common chronic infectious diseases in the world, and poses a risk to people during their entire lives. The most common cause of pain and tooth loss in the oral cavity is caries [2,3].

The United States Department of Health and Human Services stated that dental decay is the most widespread chronic disease in the world and affects both genders, all ages, and every race, and more than 95% of the population have or will have dental caries before they die [4]. In the United States, dental decay is a very common childhood disease, and is seven times more prevalent than hay fever and five times more prevalent than asthma. The incidence of tooth decay increases with age through adulthood [4].

When oral pH falls below the critical pH of 5.5, demineralization of tooth structure occurs. Reaching the critical pH, an acidic environment develops, resulting in loss of hydroxyl and phosphate ions by reacting with a surplus of hydrogen ions [5]. There are numerous data and scientific information on fluoride, indicating that fluoride consumption significantly reduces dental decay occurrence. The presence of fluoridated water in the US is perhaps responsible for the drop in the dental decay rate [6]. The reason behind this is that fluoride integrates into the hydroxyapatite (HA) crystals of teeth and forms fluorapatite, making them stronger and extra resistant to an acidic environment [7]. People can acquire fluoride by two methods: (1) systemically in water and (2) dietary supplements or topically from toothpaste and treatments at the dentist. According to the American Dental Association [8], obtaining fluoride both systemically and topically is paramount to good oral health. Therefore, using fluoride toothpaste is still needed, even if the local water is improved with the addition of fluoride.

Some studies categorize fluoride as a neurotoxin, favoring the removal of sodium fluoride from the world’s water supply. The United States National Research Council (NRC), in 2006, evaluated the EPA recommendations for fluoride standards and summarized that fluoride could be harmful to the human brain through both indirect and direct methods [9]. Higher concentrations of fluoride in drinking water can produce neurotoxic effects, and further research is necessary to ascertain a definitive conclusion [9]. In 2012, a published meta-analysis [9,10] examined a total of 27 research studies, where 26 concluded that a higher fluoride content in drinking water was associated with low IQ performance at a young age. In light of these findings, new alternatives to strengthening enamel or remineralization are needed.

Bioactive glass (NovaMin) or calcium sodium phosphosilicate usually refers to a glass precisely comprising 45 wt% SiO_2_, 24.5 wt% Na_2_O, 24.5 wt% CaO, and 6.0 wt% P_2_O_5_ [11]. Bioactive glass is indicated for the repair of bony defects or injuries that are considered too large to be regenerated by a natural process. One of the first successful surgical uses of bioactive glass was in the middle ear by replacing the ossicles to treat hearing loss [12]. Another application includes grafting bioactive glass into the jaw after tooth extractions, and it has also been used to dissolve and stimulate natural bone for self-repair, e.g., orthopedics and jaw applications [13,14].

GlaxoSmithKline is utilizing bioactive glass in toothpaste as an active ingredient under the commercial name NovaMin [15]. This toothpaste has been proven to repair small cavitations and lessen sensitivity in teeth. Palaniswamy et al. evaluated the remineralizing potential of NovaMin-containing toothpaste and amorphous calcium phosphate casein phosphopetide. The NovaMin-containing toothpaste showed a better early remineralization [16]. In 2019, Lin Lu Dai et al. summarized that bioactive glass is able to impede the growth of cariogenic bacteria and initiate remineralization by developing apatite on the surface of demineralized dentin and enamel [17]. Bioactive glass has a principal mechanism for dental decay management, which includes preventing or minimizing demineralization by promotion of remineralization and antibacterial effects on cariogenic bacteria [18]. From the American Ceramic Society (ACerS), Peter Wray stated that the United States has not approved selling bioactive glass (NovaMin) as an active ingredient in toothpaste [19]. The lack of bioactive glass in the formulation of Sensodyne’s Repair & Protect toothpaste sold in the US is surprising as bioactive glass included in toothpaste formulations sold in other countries demonstrated success in occluding exposed dentinal tubules which cause tooth sensitivity. In 2021, the first fluoride-containing bioactive glass (BioMinF) toothpaste was approved by the FDA and became available in the US [20]. The purpose of this study was to evaluate and compare the effect of fluoride and/or bioactive glass (NovaMin) on hydroxyapatite discs in the remineralization process.

## 2. Materials and Methods

### 2.1. Specimen Preparation and Treatment

A total of 22 hydroxyapatite (HA) discs (“Ca_5_(PO_4_)_3_(OH)”) were included in the study. The original HA discs were used as a control group. The discs were acid etched with 40% phosphoric acid for one minute, then immediately immersed and rinsed with distilled water for 30 s. Further, the 20 etched HA discs were divided into four groups. Each group had five etched HA discs and they were treated with four different toothpaste as follows:

Group 1: fluoride effect, Sensodyne toothpaste with fluoride (Sensodyne^®^ toothpaste, GSK, Mississauga, ON, Canada). Group 2: fluoride and bioactive glass effect, Sensodyne toothpaste with fluoride and NovaMin (Sensodyne^®^ Repair & Protect with NovaMin^®^ toothpaste, GSK, Canada). Group 3: no fluoride or bioactive glass effect, Tom’s toothpaste which lacks fluoride and NovaMin (Tom’s Fluoride-Free Natural Toothpaste, Tom’s of Maine, Kennebunk, ME, USA). Group 4: bioactive glass effect, Tom’s and NovaMin toothpaste which is a mixture of Tom’s Fluoride-Free Toothpaste 0.950 g and SYLC Bio-Active Glass prophy powder 0.050 g (Sylc, Denfotex Research Ltd., London, UK) (Table 1).

The treatment application was repeated once/day for 15 days. The toothpaste was applied on the discs using a clean micro brush (Benda Micro Applicator, Centrix, Shelton, CT, USA) for two minutes each day and rinsed thoroughly with distilled water for 30 s.

### 2.2. Scanning Electron Microscopy (SEM) Analysis

The Nova NanoSEM™ scanning electron microscope 430 was used (Nova Nano 430, FEI, Hillsboro, OR, USA) to examine the surface morphology of the discs. The control HA disc, the acid-etched HA disc, and the acid-etched and toothpaste-treated HA discs were mounted on a metal stub using an adhesive tape and then coated with carbon for analysis. SEM images were captured at various magnifications from random chosen spots on the HA discs.

### 2.3. X-ray Photoelectron Spectroscopy Analysis (XPS)

The chemical composition of the deposited films was studied using X-ray photoelectron spectroscopy (XPS) with an ESCALAB 250Xi instrument equipped with a monochromatic aluminum anode as the X-ray source. Survey scans were initially acquired from the surface of the deposited film with an electron pass energy of 50 eV, a step size of 0.5 eV, and a 900 μm diameter spot size. High-resolution scans for detailed core-level peak analysis were performed at an electron pass energy of 25 eV and an energy step size of 0.1 eV. The binding energy values were referenced to adventitious C 1s peak position at 284.6 eV.

## 3. Results

**Figure 1:**
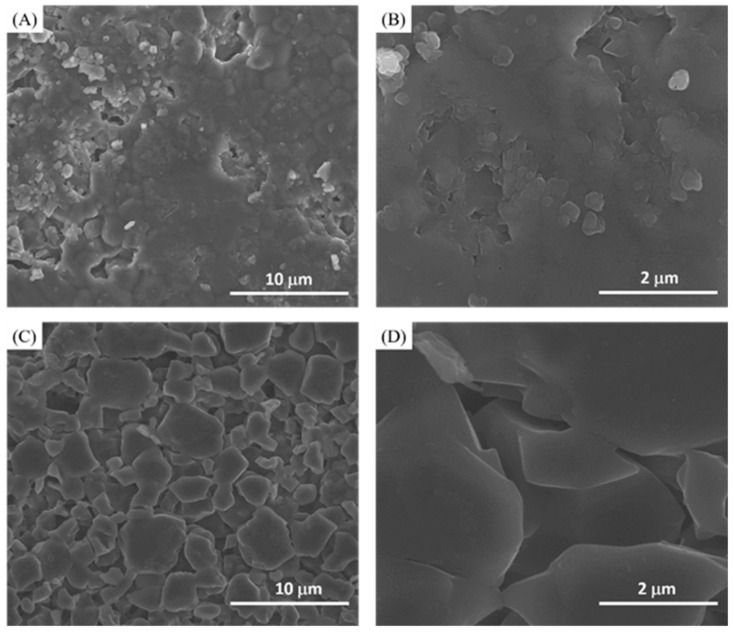
(**A**) Lower magnification and (**B**) higher magnification of HA control disc. (**C**) Lower magnification and (**D**) higher magnification of 40% phosphoric acid-etched HA discs.

All the samples were examined before and after toothpaste treatment using SEM. Figure 1 showed the morphologies for control discs (Figure 1A,B) and the acid etched discs (Figure 1C,D). The control discs showed a regular solid condensed surface with very minimal spaced out particles and crystals (Figure 1A,B). After etching, the discs showed an irregular surface morphology, with spaced out crystals, that are considered to be a typical characteristic of a demineralized enamel structure (Figure 1C,D).

**Figure 2:**
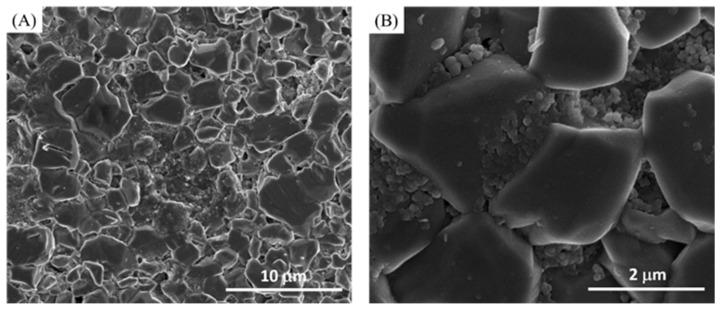
(**A**) Lower magnification and (**B**) higher magnification of Sensodyne with fluoride toothpaste-treated discs (group 1).

**Figure 3:**
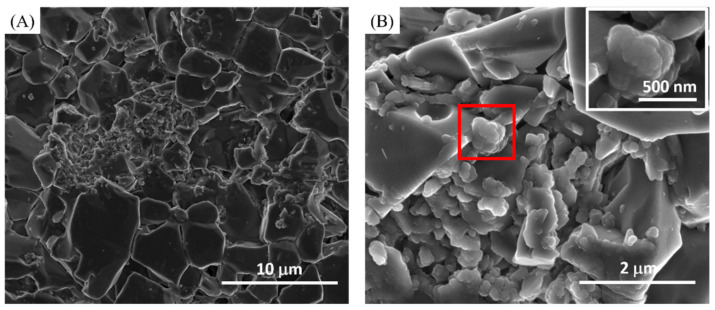
(**A**) Lower magnification and (**B**) higher magnification of Sensodyne with fluoride and NovaMin toothpaste-treated discs (group 2). The fused granules are enlarged in the insert image.

Different toothpastes were applied on the etched discs. For the fluoride effect (group 1), Sensodyne with fluoride toothpaste was used to treat etched discs. SEM revealed abundant and relatively small granules filling the spaces between the HA etched crystals (Figure 2). On the other hand, Sensodyne with fluoride and bioactive glass (NovaMin) (group 2)-treated etched discs demonstrated relatively fewer granules filling the spaces between the etched HA crystals (Figure 3). However, the granules that were fused together (coalesced) formed larger granules compared with the fluoride-containing toothpaste (group 1). The fused granules resembled a honeycomb shape that had one end attached to the HA crystal (Figure 3B inserted image).

**Figure 4:**
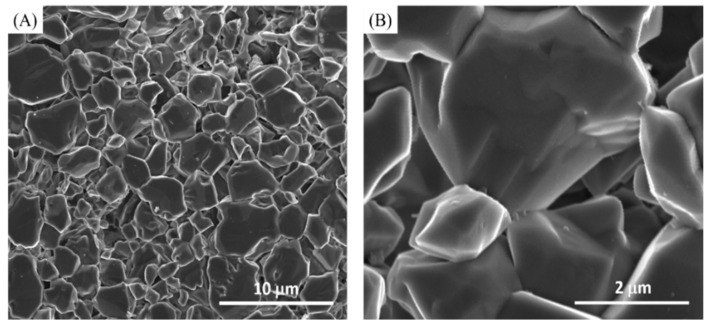
(**A**) Lower magnification and (**B**) higher magnification of Tom’s with no fluoride or NovaMin toothpaste-treated discs (group 3).

Figure 4 demonstrates Tom’s toothpaste (group 3), which has no fluoride or NovaMin content, and has no formation of granules between the HA crystals. This observation was expected due to the lack of fluoride and NovaMin in these samples. HA crystals were spread apart, similar to the appearance of the acid-etched group without any toothpaste treatment (Figure 1C,D).

**Figure 5:**
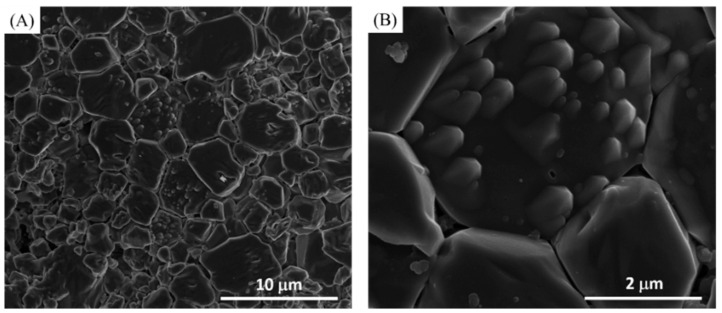
(**A**) Lower magnification and (**B**) higher magnification of Tom’s with NovaMin toothpaste-treated discs (group 4).

Lastly, bioactive glass (NovaMin) added in Tom’s toothpaste showed unique characteristics and features (group 4, Figure 5). The etched HA crystals grew noticeably closer to each other, forming granules between the crystals in a manner comparable to the fluoride-containing (group 1, Figure 2) and fluoride with bioactive glass-containing (NovaMin) (group 2, Figure 3) toothpastes. Furthermore, there was a change and/or an outgrowth in the morphology of the etched HA crystals, denoting significant signs of remineralization (Figure 5B).

**Figure 6:**
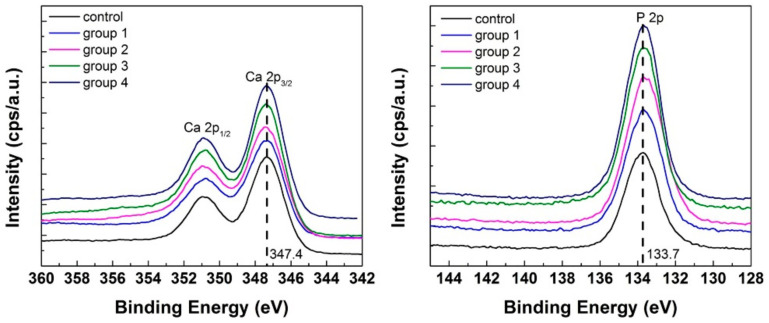
High-resolution XPS scans for Ca 2p and P 2p core levels.

The discs were analyzed using XPS investigations. The atomic percentage of each element obtained from survey scans is shown in Table 2 for control HA and toothpaste-treated etched discs. The control HA discs showed mainly O^2−^, Ca^2+^, and P^5+^ elements, and Na^+^ and Si^4+^ with lower atomic percentages. The XPS results of all toothpaste-treated etched discs showed main elements of O^2−^, Ca^2+^, P^5+^, Na^+^, and Si^4+^. S^6+^ was an additional element for Sensodyne with fluoride-treated discs (group 1), and N^5+^ and S^6+^ for Sensodyne with fluoride and bioactive glass (NovaMin)-treated discs (group 2). Additionally, N^5+^ was detected from Tom’s toothpaste with neither fluoride nor NovaMin (group 3) and Tom’s toothpaste with NovaMin (group 4). The atomic ratio of Ca/P was 1.62 for the control; 1.44 for Sensodyne with fluoride (group 1); 1.65 for Sensodyne with fluoride and NovaMin (group 2); 1.90 for Tom’s with no fluoride or NovaMin (group 3); and 1.72 for Tom’s with NovaMin (group 4) discs. High-resolution scans for core-level regions Ca 2p and P 2p are displayed in Figure 6. All spectra were similar. The binding energy of Ca 2p_3/2_ was 347.4 eV and P 2p was 133.7 eV for all the groups.

## 4. Discussion

Dental caries progression occurs due to numerous cycles of demineralization and remineralization [21]. There are several methods which can interfere with this ongoing process to reverse or halt progression of the lesion. Remineralization is considered a biological repair process for non-cavitated lesions, and relies heavily on phosphate and calcium ions, in addition to fluoride, to rebuild a new surface on existing crystals in lesions remaining after demineralization. The remineralized crystals are much less soluble than the original tooth structure; therefore, the crystals are acid resistant [21]. For decades, fluoride was incorporated into toothpaste to improve the process of remineralization. Currently, researchers and laboratories are investigating ingredients other than fluoride to incorporate into toothpaste to assist in the mineralization process of dental caries.

Bioactive glass was invented in the early 1970s [22]. These glasses are silica-based, very biocompatible materials, and exhibit bone bonding as a result of the surface reactive silica, calcium, and phosphate groups. Treatment of dentin hypersensitivity by implementing bioactive glass (NovaMin) has shown statistically significant and encouraging clinical results [23,24]. In 2006, Zero demonstrated that when NovaMin was exposed to saliva or water, an immediate reaction occurred by releasing billions of minerals, such as calcium, silica, phosphorous, and sodium, that helped to boost the process of remineralization [25]. In this study, HA discs were used to mimic the enamel layer of natural teeth, where approximately 95% to 98% of enamel contains phosphate and calcium ions that form strong hydroxyapatite crystals [26,27].

The HA discs were etched with 40% phosphoric acid to initiate enamel demineralization. Tom’s toothpaste with no fluoride or NovaMin showed no signs of remineralization, which was anticipated due to the lack of pivotal constituents like fluoride and phosphate. Primarily, Tom’s toothpaste with no fluoride or NovaMin is mainly composed of hydrated silica for cleaning/polishing and sodium bicarbonate for mouth freshness [28]. Hamza et al. studied the abrasivity and cleaning efficacy of toothpastes. Hydrated silica had higher abrasivity compared with alternative abrasives [29]. This might explain our findings where the morphologies between Tom’s toothpaste and etched HA discs were similar (Figure 1C,D and Figure 4).

Interestingly, Sensodyne with fluoride and NovaMin toothpaste formed larger granules (honeycomb shape) that were found at the inter-crystal gaps versus Sensodyne with fluoride alone, for which the granules were smaller in size. We postulate that the addition of NovaMin to fluoride toothpaste contributed a synergistic effect. This is in agreement with other studies [30,31]. Burwell et al. reported that fluoride alone did not attribute to remineralization effectively, whereas incorporated NovaMin with fluoride showed an improved hardness after remineralization [31].

Commercially available NovaMin-containing toothpastes typically have fluoride as an additional ingredient. However, the amount of fluoride from sodium monofluorophosphate contained in NovaMin toothpaste was questionable [32,33]. As the toothpastes are applied on the teeth, the fluorides are possibly washed away through salivary flow. The sodium from NovaMin is ion exchanged with hydrogen ions. The calcium and phosphate are released from NovaMin into the surrounding environment. As pH temporarily increases, the released calcium and phosphate ions form a Ca-P layer on the tooth surface and crystallize into hydroxycarbonate apatite [31,34]. Additionally, fluoride is incorporated into the bioactive glass instead of creating an additional ingredient [33,35,36]. Shah et al. studied the bioactivity of bioactive glass containing different percentages of fluoride in comparison with bioactive glass. Fluorite was formed when the fluoride content was increased in the glass. This did not favor apatite formation [35]. Farooq et al. compared the remineralization potential of BiominF toothpaste which contained fluoride and a high level of phosphate to NovaMin toothpaste. The results showed that BiominF had better remineralization potential [33].

In this study, the authors wanted to determine the impact of bioactive glass alone (NovaMin) on HA discs without any potential fluoride interaction. Adding bioactive glass (NovaMin) powder into distilled water did not work because the powder precipitated instead of dissolving into the solution. Therefore, the idea of mixing 0.050 g bioactive glass (NovaMin) powder into 0.950 g Tom’s Fluoride-Free Toothpaste was developed. The results demonstrate that bioactive glass (NovaMin) interacted with the hydroxyapatite crystals, causing an alteration of their morphology, and an outgrowth occurred which possibly minimized the inter-crystal gaps. This is thought to be an indication of a faster remineralization process compared to fluoride or fluoride and NovaMin combined. Mony et al. reported that NovaMin was effective in remineralization and could be an alternative material to fluorides [37]. However, Körner et al. reported their findings in which fluoride toothpaste showed increased remineralization compared with a hydroxyapatite toothpaste or additional bioactive glass slurry treatment [38].

Further, the discs were analyzed using XPS (Table 2) to determine if there was a change in surface composition after treatment. The control discs showed the main composition as hydroxyapatite (Ca_5_(PO_4_)_3_(OH) with Na^+^ and Si^4+^. Different toothpastes were applied on the etched discs and were analyzed. The Sensodyne with fluoride-treated discs (group 1) showed a new layer with lower concentrations of Ca^2+^ and Na^+^ compared with control discs, and Si^4+^ and S^6+^. The Ca^2+^ and P^5+^ showed higher concentrations with the Sensodyne with fluoride and NovaMin toothpaste (group 2) compared with the Sensodyne with fluoride toothpaste (group 1). The additional observed Na^+^, Si^4+^, and S^6+^ could be from the ingredients of the toothpastes. Theoretically, the hydroxyapatite composition showed a Ca/P ratio of 1.66 and the control HA discs in this study showed a similar Ca/P ratio of 1.62. After acid etching and toothpaste treatments, the new surface layer showed a Ca/P ratio of 1.44 for the Sensodyne with fluoride discs (group 1), and 1.65 for the Sensodyne with fluoride and NovaMin discs (group 2). This might indicate a synergistic effect between fluoride and NovaMin.

However, the etched discs treated with Tom’s toothpaste, with neither fluoride nor NovaMin (group 3), showed decreased Ca^2+^ and P^5+^ but increased Na^+^ compared with control discs. The Na^+^ and Si^4+^ could also be ingredients from Tom’s toothpaste. The decreased Ca^2+^ and P^5+^ could be the result of the hydrated silica contained in Tom’s toothpaste which function as an abrasive to clean and polish teeth. There was a lack of fluoride and bioactive glass active components, which could initiate remineralization, in Tom’s toothpaste. These results are in agreement with SEM findings where the surface morphology of Tom’s-treated discs (Figure 4) showed no signs of remineralization but an irregular surface morphology and spaced out crystals, which were very similar to the appearance of etched discs without treatment (Figure 1C,D). Finally, the Tom’s toothpaste with NovaMin (group 4) demonstrated the role of NovaMin in the remineralization process. The Ca^2+^ and P^5+^ showed higher percentages than in the etched discs treated with the other three toothpastes. The ratio of Ca/P was 1.72 for the etched discs treated with Tom’s toothpaste with NovaMin, which was higher than all the other toothpaste groups. Based on SEM and XPS results, Tom’s toothpaste with NovaMin might demonstrate a faster remineralization among all the test groups. Although the ratio of Ca/P was changed as well as surface morphologies after toothpaste treatments, the binding energies of Ca and P from XPS high-resolution images after toothpaste treatments were the same as for the hydroxyapatite control group [39]. This might indicate that the surface chemistry remained similar to that of hydroxyapatite.

Finally, the US Food and Drug Administration (FDA) verified that with an approved amount of fluoride compounds, fluoride does help to prevent dental cavities when added to toothpastes [40]. Taking into account the numerous studies on bioactive glass (NovaMin) and its positive effect on hydroxyapatite (tooth enamel) reformation, bioactive glass (NovaMin) can serve as an alternative ingredient in remineralization.

## 5. Conclusions

Within the limitations of this comparative in vitro study, the conclusions are: (1) Bioactive glass (NovaMin) alone exhibited promising remineralization capabilities compared with a combination of fluoride and bioactive glass or just fluoride; (2) bioactive glass with fluoride seemed to potentiate the effect of fluoride alone; and (3) the absence of both bioactive glass and fluoride offered no remineralization benefit. Future studies could include examining the rate of remineralization as a function of time and frequency of application.

## Figures and Tables

**Table 1:** Four groups of toothpaste and their ingredients.

Groups	Toothpaste	Ingredients
Group 1: 1–5 HA etched discs	Sensodyne with fluoride	Active ingredient: Stannous fluoride, 0.454% (0.15% *w*/*v* fluoride ion). Antihypersensitivity, anticavity. Inactive ingredients: Hydrated silica, glycerin, polyacrylic acid, pentasodium triphosphate, sodium saccharin, sodium lauryl sulfate, titanium dioxide, PEG-8, cocamidopropyl betaine, flavor.
Group 2: 6–10 HA etched discs	Sensodyne with fluoride and NovaMin	Medical ingredients: Calcium sodium phosphosilicate (Novamin) 5.0% *w*/*w*, sodium monofluorophosphate 0.788% *w*/*w* (fluoride 0.104% *w*/*w*). Nonmedical ingredients: (Alpha) carbomer, DL-Limonene, glycerin, linalool, mint flavor, PEG-8, silica, sodium lauryl sulfate, sodium saccharin, titanium dioxide.
Group 3: 11–15 HA etched discs	Tom’s with no fluoride or NovaMin	Arginine bicarbonate, calcium carbonate, hydrated silica, xanthan, peppermint oil, benzyl alcohol, sodium bicarbonate, sodium lauryl sulfate, titanium dioxide, water, gum, xylitol, sorbitol.
Group 4: 16–20 HA etched discs	Tom’s with NovaMin	Tom’s: Arginine bicarbonate, calcium carbonate, hydrated silica, sodium bicarbonate, sodium lauryl sulfate, peppermint oil, benzyl alcohol, sorbitol, titanium dioxide, water, xanthan gum, xylitol. Bioactive glass (NovaMin): The powder ingredient is calcium sodium phosphosilicate. The glass is composed of 45 wt% SiO_2_, 24.5 wt% Na_2_O, 24.5 wt% CaO, and 6.0 wt% P_2_O_5_.

**Table 2:** The atomic composition of control, etched discs, and toothpaste-treated discs obtained from XPS survey spectra. Cls was excluded from the estimations due to surface contamination. O 1s peak might also contain a contribution from adsorbed water.

Group	Samples	O 1s	Ca 2p	P 2p	Na 1s	Si 2s	N 1s	S 2p	Ca/P
Control	Control	59.48	20.05	12.41	5.06	3.00			1.62
Group 1	Sensodyne with fluoride	62.66	18.30	12.71	1.18	4.56		0.59	1.44
Group 2	Sensodyne with fluoride and NovaMin	60.13	21.39	13.00	2.97	1.27	0.51	0.73	1.65
Group 3	Tom’s with no fluoride or NovaMin	55.7	18.68	9.82	9.27	2.29	4.24		1.90
Group 4	Tom’s with NovaMin	57.69	24.32	14.11	0.66	2.68	0.54		1.72

## Data Availability

Not applicable.
